# A quiet gap: why private equity in primary care remains under-researched in the global south, and why that matters

**DOI:** 10.3389/fpubh.2026.1953760

**Published:** 2026-09-30

**Authors:** Marc Kitten, Chiara Berardi

**Affiliations:** Global Business School for Health, Faculty of Population Health Sciences, University College London, London, United Kingdom

**Keywords:** financialisation, global south, health-system governance, primary health care, private equity, public-private partnerships, stewardship, primary care

## 1 Introduction

Private equity (PE) has become a material feature of primary health care (PHC) delivery across the Global North. In the United States, the number of PE-owned physician-practise sites rose more than six-fold between 2012 and 2021 (1), and these acquisitions have been associated with higher prices and spending (2, 3). By 2024, an estimated 6.5% of physicians worked in PE-owned practises, up from 4.5% in 2022, with PE involvement exceeding 30% in some specialties (4). In the United Kingdom, the special-situations investor Aurelius acquired LloydsPharmacy in 2021 for £477m; the network entered liquidation in 2024, and its more than 1,300 NHS-contracted pharmacies were sold or closed (5). In France, the financialisation of medical biology has attracted regulatory and scholarly scrutiny (6). Comparable consolidation has occurred in continental European dentistry, where PE-backed groups such as Colosseum and EDG drove acquisitions in the late 2010s (7).

Most of these providers operate within public reimbursement frameworks. PE ownership of primary care therefore constitutes, in effect, a public-private arrangement, yet its governance terms remain poorly specified. Two questions follow: how do public reimbursement decisions and private financial strategies jointly shape market outcomes, and on what terms should that interaction be governed? The Global North already offers evidence on both. In much of the Global South the same capital is arriving later and in a different form, creating a narrow window in which that evidence can inform policy prospectively rather than retrospectively.

## 2 A de facto public-private partnership

Stewardship denotes the state's overarching responsibility for the health system, including the private actors within it (8, 9). Governance refers to the mechanisms through which that responsibility is exercised, including transparency, information, and accountability (10). Although the terms are often used interchangeably in policy practise, the distinction is operationally consequential: a ministry may hold a clear stewardship mandate while lacking the governance instruments to act on it, and diagnosing which is absent determines whether the remedy is legal authority or administrative capacity. PE ownership tests that capacity directly, introducing financial actors whose structure and conduct fall outside conventional provider oversight. In the US, financial incentives embedded in payment policies play an outsized role in shaping PE strategies and their relationship with healthcare outcomes (11).

The most comprehensive synthesis is Borsa et al.'s (12) review of 55 studies across eight high-income countries, which found predominantly mixed or adverse effects on quality and market outcomes, with increased market consolidation and, in some settings, higher prices. A subsequent review reaches similar conclusions for the United States (13). The financialisation literature identifies the mechanism linking ownership to these outcomes: finance enters health care through ownership structures and instruments that can divert value from service delivery towards investors (14, 15).

Whether private capital should be admitted to PHC at all remains a legitimate and contested question (16–19), and one this piece does not attempt to settle. Our starting point is empirical rather than normative: PE is already present in PHC across the Global North, and is being written into universal-coverage frameworks. Our argument is correspondingly narrower and conditional. Where that capital is already present, the terms on which it operates are consequential and at present poorly specified. When a public payer reimburses a PE-owned provider, it contracts with a counterparty whose financial structure it neither observes nor governs. Present contracting frameworks are organised around services, volumes, and quality metrics, and are largely silent on ownership, leverage, investor horizon, and exit.

## 3 What the Northern evidence shows

Treating private equity ownership as a uniform category obscures relevant variation. Where studies disaggregate by deal type and setting, outcomes depend on three factors: the capital structure used to acquire the asset, the investor's time horizon, and the strength of the surrounding governance (20, 21). Highly leveraged roll-ups that concentrate local market power are associated with higher prices (20), and post-acquisition reductions in clinical staffing are associated with poorer patient outcomes (22). Growth investment financed with less debt and debt-heavy buyouts carrying greater insolvency risk are distinct ownership forms with distinct risk profiles, which aggregate estimates cannot capture. The distinction carries into Southern policy: where growth equity predominates, the priority is disclosure and quality monitoring; where leveraged buyouts appear, solvency and continuity of service also require attention.

The public payer is itself an actor in the risk environment of the providers it contracts with, a role existing stewardship frameworks rarely acknowledge: reimbursement policy shapes the conditions under which leveraged providers operate. The Aurelius acquisition of LloydsPharmacy was priced against a community-pharmacy reimbursement environment that subsequently tightened, and the chain entered insolvency in 2024, with reduced reimbursement identified among the pressures on its capital structure (5). In France, successive reductions to the medical-biology fee schedule have compressed the margins on which leveraged consolidation depends (6).

Opaque ownership through shell companies and layered holding structures has repeatedly impeded oversight (15), though this evidence base is drawn overwhelmingly from high-income settings.

## 4 The quiet gap in the Global South

The asymmetry in the published literature mirrors an asymmetry in the underlying deal activity. Of approximately 20,000 PE health care transactions recorded between 2000 and 2026 Q1, 90% targeted the Global North (23). These figures cover health care as a whole rather than primary care specifically, but the trajectory is indicative of how PE in Southern PHC is likely to develop. The Global South has no evidence base equivalent to the Northern literature on PE in health care reviewed above.^1^ Of approximately 232 articles on PE in health care indexed in PubMed since 2000, 84% concern the United States, 7% Europe, and only 3 address Global South settings: one on Turkey (26) and two on Australia (27, 28).

Recorded PE activity in the Global South's health care is not only lower than in the North but different in kind. It is characterised less by the leveraged consolidation of fragmented primary care than by development-finance-institution co-investment, impact-oriented growth equity, and greenfield clinic and hospital construction, as documented in PE fund investment in Turkey (26) and in the financialisation of insurance and hospital provision in Brazil (29). These blended-finance arrangements are partly structured to mitigate the risks of the conventional leveraged PE model. However, as these markets mature and commercial capital displaces concessional and development finance, the leveraged models now prevalent in the Global North are likely to follow. The Global South is not homogeneous in this respect. Some countries pair a universal system with a large private sector and established regulators, such as Brazil. Others remain substantially donor-dependent with thinner oversight capacity, such as Nigeria. Others still, like India and Indonesia, sit between these poles. Exposure to PE and the capacity to govern it therefore differ markedly, and the priorities below require calibration to each setting. Several systems in the Global South are currently designing universal-coverage PPP frameworks, yet have not addressed how contracting with a private partner that is a finite-horizon, leverage-financed vehicle distributes risk and reward between the state and the private sector and shapes its incentives. Debt is central to that distribution: where a special-purpose vehicle carries the borrowing and the state underwrites it explicitly or implicitly, downside risk returns to the public balance sheet while returns accrue privately.

## 5 Stewardship before scale: from reactive to anticipatory governance

The Northern evidence indicates with some consistency where public-payer attention should be directed, aligning with three established principles of health-system governance (10, 30).

The first is transparency. Publicly traded providers already disclose ownership and capital structure under listing rules, but privately held PE vehicles often face no equivalent requirement, which is where oversight is weakest. Mandatory annual disclosure of beneficial ownership and capital structure should therefore be a standing condition of public reimbursement for all contracted providers, rather than an investigation undertaken after a provider fails (15). The second is intelligence and information. Payers unable to assess a provider's capital structure cannot anticipate which providers a reimbursement change will place under strain; since reimbursement decisions often precipitate that strain, solvency implications should be assessed ex ante within the policy process rather than ex post. The third is accountability. Effects on cost and quality emerge only with a lag, whereas deteriorating workforce conditions, including staffing levels, clinical autonomy, and throughput pressure, are often the earliest observable indication of how an ownership model operates and should be measured early (22). Equity belongs in the same frame: obligations concentrated in profitable segments or locations can draw provision away from underserved areas, so distributional effects across the system warrant monitoring alongside cost and quality.

These are established tools of market shaping, including beneficial-ownership registers, change-of-control notification, contract design, and professional governance, applied to a relatively recent ownership form. Several Northern regulators have begun to adopt them (31). Their particular value in the Global South is that they can be introduced while the sector remains small enough to shape. Implementation must nonetheless match administrative capacity: where a ministry cannot itself verify beneficial ownership, disclosure can be routed through company registries or financial regulators, or made a condition of licencing and of development-finance co-investment, rather than becoming a new ministerial function; where provider accounts are not standardised, a minimal reporting template for the largest contracted providers is more tractable than full solvency assessment. Each country will need to designate which authority is responsible for verifying disclosure and for applying sanctions where requirements are breached. Development-finance institutions co-invest alongside PE across Southern health care and are therefore a natural point of leverage, since disclosure and continuity-of-service covenants can be embedded in the capital itself. That they have largely not used it reflects competition with commercial capital for the same transactions, which makes unilateral covenants costly; coordinated adoption is the more plausible route.

## 6 Discussion

Governments do not adopt governance arrangements merely because evidence supports them. Decisions to admit private capital into PHC are shaped by fiscal pressure, borrowing conditions, and investor lobbying, while disclosure obligations impose visible costs on investors and deliver diffuse, deferred benefits. Anticipatory governance is therefore likeliest to take hold where it is embedded in financing conditions at entry rather than legislated afterwards. Systems lacking these stewardship and governance structures will face the greatest difficulty. The task is to build that capability into PHC market design in advance, rather than in reaction to consolidation that has already occurred. The gap in the literature is thus not only an absence to be regretted but an opportunity to govern the capital, and not only the service, before the terms are settled by default.

Three objections merit acknowledgement. Transparency presupposes baseline data, so where PHC performance data are absent, ownership disclosure should be sequenced alongside basic provider reporting rather than ahead of it. Disproportionate requirements may deter capital that Southern systems need for UHC expansion, which argues for obligations scaled to provider size and contract value. And regulatory capture is a genuine risk where PE firms are the main source of expertise on their own structures, favouring regional pooling of that expertise over bilateral reliance.

Several limitations remain. The effectiveness of these priorities will depend on each setting's institutions and regulatory capacity. The Global South evidence base outlined here, drawn from PubMed, is a preliminary assessment rather than a systematic review. PitchBook may also understate deal volume, since smaller transactions are less likely to be captured, though it remains among the most comprehensive sources available. Future research should track early adopters of disclosure and information-sharing requirements in Global South primary-care PPPs, and should examine empirically whether anticipatory rather than reactive governance produces measurable changes in outcomes for patients, workers, and public payers.

## Footnotes

1 Following (24), “Global South” denotes a geopolitical and geo-historical category, broadly the regions of Latin America, Asia, Africa, and Oceania, rather than a World Bank income band. It overlaps with, but is not coextensive with, the low- and middle-income country (LMIC) classification (25). Several high-income economies are conventionally treated as Southern, such as the Gulf states and Singapore, while the status of large emerging powers such as China and India is contested. We use Global North and Global South as the consistent frame and reserve income-band terms for granularity within the South.
